# The central role of transfer RNAs in mistranslation

**DOI:** 10.1016/j.jbc.2024.107679

**Published:** 2024-08-16

**Authors:** Dominik B. Schuntermann, Mateusz Jaskolowski, Noah M. Reynolds, Oscar Vargas-Rodriguez

**Affiliations:** 1 Department of Biology, Institute of Molecular Biology and Biophysics, Zurich, Switzerland; 2 School of Integrated Sciences, Sustainability, and Public Health, University of Illinois Springfield, Springfield, Illinois, USA; 3 Department of Molecular Biology and Biophysics, University of Connecticut Health Center, Farmington, Connecticut, USA

**Keywords:** tRNA, genetic code, mistranslation, protein synthesis, aminoacyl-tRNA synthetases, ribosome, editing, post-transcriptional modifications

## Abstract

Transfer RNAs (tRNA) are essential small non-coding RNAs that enable the translation of genomic information into proteins in all life forms. The principal function of tRNAs is to bring amino acid building blocks to the ribosomes for protein synthesis. In the ribosome, tRNAs interact with messenger RNA (mRNA) to mediate the incorporation of amino acids into a growing polypeptide chain following the rules of the genetic code. Accurate interpretation of the genetic code requires tRNAs to carry amino acids matching their anticodon identity and decode the correct codon on mRNAs. Errors in these steps cause the translation of codons with the wrong amino acids (mistranslation), compromising the accurate flow of information from DNA to proteins. Accumulation of mutant proteins due to mistranslation jeopardizes proteostasis and cellular viability. However, the concept of mistranslation is evolving, with increasing evidence indicating that mistranslation can be used as a mechanism for survival and acclimatization to environmental conditions. In this review, we discuss the central role of tRNAs in modulating translational fidelity through their dynamic and complex interplay with translation factors. We summarize recent discoveries of mistranslating tRNAs and describe the underlying molecular mechanisms and the specific conditions and environments that enable and promote mistranslation.

Translation of genetic information into proteins is a pillar of life. At the center of translation are transfer RNAs (tRNAs), the small non-coding RNAs responsible for facilitating the decoding of information from messenger RNAs (mRNAs) into proteins (1, 2). During translation, tRNAs interact with multiple factors, including tRNA-modifying enzymes, aminoacyl-tRNA synthetases (aaRSs), ribosomes, and elongation factors (EF-Tu in bacteria and EF1A in archaea and eukarya). Faithful and effective interactions between tRNAs and their interacting partners are indispensable for accurately translating the genetic code. Due to the complexity of these interactions, errors often occur, causing the insertion of amino acids in the wrong position of protein sequences (3, 4, 5). The frequency of translational errors, or mistranslation, is estimated at one mistake per every 10^3^-10^4^ translated codons in standard growth conditions (4, 6, 7, 8). However, different factors, such as environmental conditions (*e.g.*, nutritional limitation) and growth stages (*e.g.*, fast *versus* stationary growth), modulate the mistranslation rate (9). In addition to their inherent propensity for mistranslation, organisms can encode dedicated factors or mechanisms to mistranslate their genetic codes deliberately. In this review, we discuss the fundamental aspects of protein synthesis and the factors that modulate the accurate translation of genomic information, focusing on the central role of tRNAs in translational fidelity. We also review recent discoveries of noncanonical tRNAs that challenge our understanding of the genetic code and the biological implications of mistranslation.

### tRNA structure and function

tRNAs comprise 70 to 100 nucleotides that form a conserved cloverleaf-like secondary structure (Fig. 1*A*). Five distinct arms define the structure: 1) the acceptor-, 2) anticodon-, 3) TΨC- (where T and Ψ indicate ribothymidine and pseudouridine, respectively), 4) variable-, and 5) dihydrouridine (D)-arms (10, 11, 12, 13, 14). A network of conserved canonical and noncanonical base pairing induces the formation of an L-shaped tertiary structure through the stacking of the TΨC -arm (also known as the T-arm) onto the acceptor stem and the folding of the anticodon stem-loop onto the D-arm (Fig. 1*B*). The structure of tRNAs is also modulated by post-transcriptional modifications, which occur throughout the tRNA scaffold but are more frequently found in the D-arm, T-arm, and anticodon loop (15). tRNA modifications can be specific to a tRNA or universally conserved. They can play different roles in tRNA biology, including structural stability, aminoacylation, and mRNA decoding. Across the tRNA L-shaped structure exists an RNA code that defines the specific interactions with tRNA’s binding factors. The sequence, length, and architecture of tRNAs provide the framework for specific interactions with their different interacting partners, which define their specificity, identity, and function (16, 17, 18).

**Figure 1:**
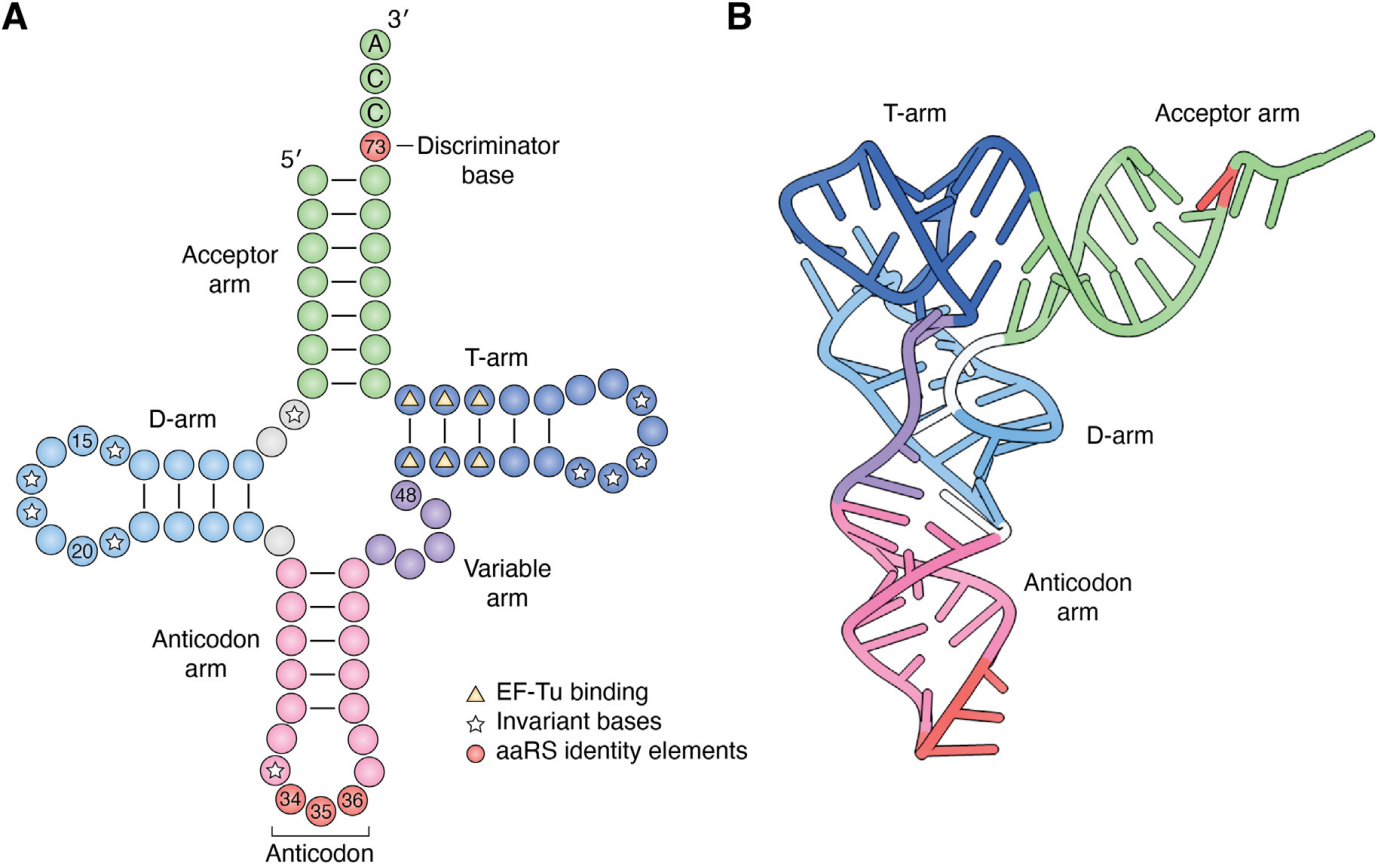
**tRNA structure.***A*, cloverleaf-like secondary conformation of tRNAs. tRNAs consist of an acceptor arm (*green*), D-arm (*light blue*), anticodon-arm (*pink*), variable arm (*purple*), and T-arm (*dark blue*). The discriminator base N73 and anticodon bases N34-N36 (*red*) serve as universally conserved aaRS identity elements. Invariant bases across tRNAs are highlighted with a star and important nucleotides for EF-Tu binding with a *triangle*. *B*, tertiary L-shape structure of tRNA^Phe^ [PDB: 1EHZ].

Each organism is expected to encode a set of tRNA genes that enable the translation of its genetic code. The number of tRNA genes is unique to each organism and varies greatly among species. For example, humans encode over 400 tRNA genes, while *Saccharomyces cerevisiae* harbors 275 (19). Due to the degeneracy of the genetic code and translation requirements, the tRNA set of an organism includes tRNAs with different anticodons that carry the same amino acid (tRNA isoacceptors) and tRNAs with the same anticodon but different body (tRNA isodecoders) (1).

The primary function of tRNAs is to supply the ribosome with amino acid substrates during mRNA-templated protein synthesis. During the reiterative translational process, tRNAs are subjected to cycles of aminoacylation and delivery to the ribosomes (Fig. 2). tRNA aminoacylation, which is catalyzed by aaRSs, occurs in two steps. First, the amino acid is “activated” with ATP to form an aminoacyl-adenylate intermediate. In the second step, the amino acid is esterified to the tRNA’s 3′-terminal adenosine (Fig. 3*A*). The resulting aminoacyl-tRNA (aa-tRNA) product is then released by aaRSs, in some cases, with the assistance of EFs (20). EF-Tu (or EF1A in archaea and eukaryotes) transports aa-tRNAs to the ribosome, where productive tRNA-mRNA (anticodon-codon) interactions are established before EF-Tu releases aa-tRNAs. The ribosome uses incoming aa-tRNA substrates to elongate the growing polypeptide chain. These steps are highly choreographed and occur with outstanding speed and reliability. Nonetheless, errors in protein synthesis are unavoidable due to the inherent error-prone nature of aaRSs, EFs, and the ribosome or to modifications or conditions that alter their specificities. Additionally, specialized translation factors that mistranslate the genetic code also exist. In the following sections, we describe the concept of mistranslation and its causes with a centric view of tRNAs.

**Figure 2:**
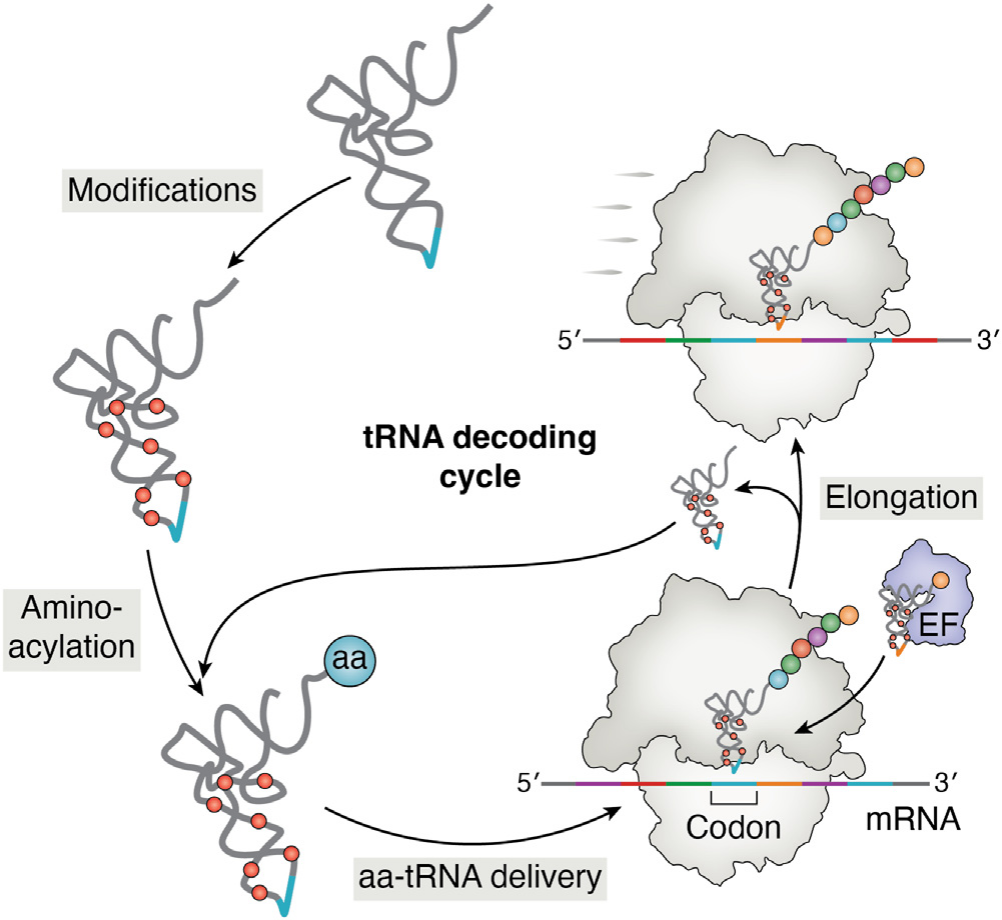
**tRNA decoding cycle.** tRNAs are post-transcriptionally modified before aminoacylation by their respective aaRSs. After aminoacylation, aa-tRNAs are delivered to the ribosome by elongation factors (EF-Tu or EF1A), where they base-pair with their corresponding codon. After codon recognition, the delivered amino acid (aa) is added to a nascent polypeptide chain by the ribosome (the elongation step). The “uncharged” tRNA enters a new cycle of aminoacylation and elongation.

**Figure 3:**
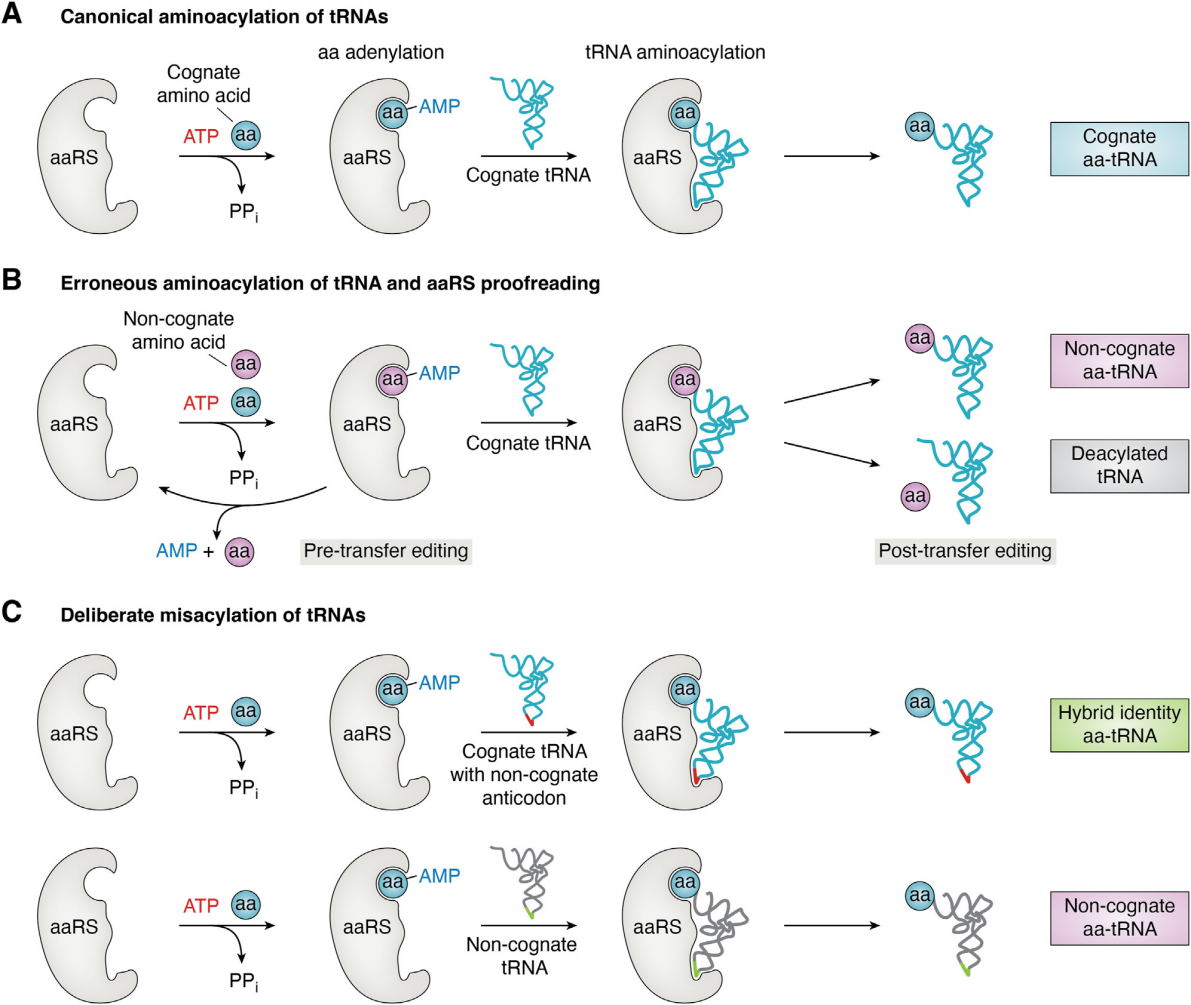
**Mechanisms of aminoacyl-tRNA synthesis.***A*, aaRSs bind their cognate amino acid (aa, *grey circle*) and ATP to produce an aminoacyl-adenylate (aa-AMP) intermediate. aaRSs select the corresponding tRNAs based on intrinsic identity elements and ligate the amino acid to form cognate aminoacyl-tRNA (aa-tRNA). *B*, aaRSs occasionally bind and activate noncognate amino acids (ncaa, *pink circle*). Some aaRSs employ pre-transfer editing to hydrolyze noncognate aa-AMP before the ncaa is transferred to a tRNA. However, pre-transfer editing is not present in all aaRSs and is insufficient to prevent the ligation of the wrong amino acid to the tRNA. aaRSs with post-transfer editing can deacylate incorrectly synthesized aa-tRNA, which results in free ncaa and tRNA. In the absence or failure of editing mechanisms, the mismatched aa-tRNA is synthesized and can participate in protein synthesis. *C*, in rare cases, an amino acid is purposely ligated to a tRNA with an anticodon that does not correspond to the amino acid. This is mediated by aaRSs with altered specificities or by the existence of tRNAs with hybrid identities. If left uncorrected, these misacylated tRNAs participate in protein synthesis. [aaRS, aminoacyl-tRNA synthetase; ATP, adenosine triphosphate; AMP, adenosine monophosphate; PP_i_, inorganic pyrophosphate; aa, cognate amino acid; ncaa, noncognate amino acid].

### Mistranslation of the genetic code

Mistranslation occurs when a codon is translated with the wrong amino acid. The direct outcome of mistranslation is the production of different protein variants from the same coding gene. The resulting protein variants may i) retain the activity of the encoded protein, ii) display altered activity (*e.g.*, reduced efficiency or changed specificity), iii) lose functionality, or iv) gain a new activity. The impact of these variants on cellular homeostasis varies based on the organism and the environmental context. Mistranslation events are generally fortuitous, caused by translation factors (*e.g.*, aaRSs and ribosomes) with error-prone specificities or whose specificities have been compromised by mutations or modifications (14, 21, 22, 23, 24, 25). Consequently, mRNAs are mistranslated stochastically with varying rates of amino acid misincorporations across protein sequences. Fortuitous mistranslation is greatly influenced and induced by cellular conditions. For example, changes in the cellular concentration of amino acids can prompt aaRSs to ligate noncognate amino acid substrates to tRNAs (26). Other environmental stresses, such as oxidation and antibiotics, can also promote mistranslation by altering the specificities of translation factors (7, 22, 27, 28). However, in some organisms, mistranslation can be genetically encoded. In these cases, dedicated translation factors or mechanisms are encoded to mistranslate the genetic code (5, 29, 30). In contrast to fortuitous mistranslation, encoded mistranslation offers a degree of specificity, targeting specific codons with a noncognate amino acid and enabling the organism to induce mistranslation selectively. Thus, the critical distinction between fortuitous and encoded mistranslation is the ability of the organism to regulate it, which helps minimize the potential adverse effects of mistranslation.

## Mistranslation prevention

### Proofreading of aa-tRNA synthesis

The failure of aaRSs to efficaciously reject noncognate amino substrates is compensated by the existence of quality control checkpoints to proofread aa-tRNA synthesis. Approximately half of all aaRSs (MetRS, LeuRS, IleRS, ValRS, SerRS, ProRS, ThrRS, LysRS, PheRS, and AlaRS) possess mechanisms to avoid or correct tRNA aminoacylation errors known as proofreading or editing. aa-tRNA editing can either prevent the attachment of the wrong amino acid to a tRNA (known as “*pre-transfer editing*”) or catalytically separate the amino acid after it is ligated to the tRNA (known as “*post-transfer editing*”) (31, 32, 33). These quality control mechanisms are implemented in two different catalytic sites. Pre-transfer editing occurs in the aminoacylation catalytic site after an incorrectly activated aminoacyl-adenylate is formed. aaRSs can selectively hydrolyze the aminoacyl-adenylate or release it from the catalytic pocket for spontaneous hydrolysis (34). In contrast to pre-transfer editing, post-transfer editing is catalyzed by specialized domains that cleave the wrongly synthesized aa-tRNA. LeuRS, IleRS, ValRS, ThrRS, PheRS, AlaRS, and ProRS have evolved editing domains to correct their aminoacylation mistakes (31, 35). Post-transfer editing can also be catalyzed by aaRS-independent aa-tRNA deacylases that usually consist of single-domain proteins (35). These enzymes are phylogenetically related to the editing domains of PheRS, AlaRS, ThrRS, and ProRS. aa-tRNA deacylases are predominantly grouped in the AlaX (related to AlaRS), DTD (d-aminoacyl-tRNA deacylases), CtdA (related to PheRS), ThrRS-ed (related to ThrRS), and INS (related to ProRS) families (35, 36, 37). Notably, freestanding deacylases display substrate specificities that do not necessarily correspond to their relationship with aaRSs. For example, the recently discovered CtdA deacylase, which is evolutionarily related to PheRS, corrects aminoacylation errors by arginyl-tRNA synthetase (ArgRS), which charges the non-proteinogenic amino acid canavanine to tRNA^Arg^ (36). Similarly, members of the INS family have evolved to correct mistakes emanating from several aaRSs other than ProRS, including AlaRS, ThrRS, LysRS, ValRS, and others (38, 39).

An interesting characteristic of editing domains is that they generally exhibit relaxed substrate specificities, particularly towards the aminoacyl moiety of their aa-tRNA substrates. This allows them to recognize different aa-tRNAs. For example, the editing domain of AlaRS hydrolyzes two distinct substrates produced by AlaRS, Ser- and Gly-tRNA^Ala^. Similarly, the editing domain of PheRS can deacylate *meta*-Tyr (*m*-Tyr) and *para*-Tyr (*p*-Tyr)-tRNA^Phe^ (27, 40), while LeuRS’s editing domain recognizes Ile- and norvanyl-tRNA^Leu^ (41, 42). This relaxed aminoacyl specificity is also observed for the editing domains of IleRS, ValRS, ProRS, and single-domain editing enzymes (31, 35). Thus, given the myriad of naturally occurring, non-coded amino acids, additional aa-tRNA substrates for editing domains may still be unidentified.

Unlike their amino acid moiety specificity, editing domains show higher selectivity towards tRNAs, helping to prevent the undesired and energetically costly hydrolysis of correctly aminoacylated tRNAs. tRNA elements play a critical role in the different mechanisms of selection and recognition. For the editing domains of PheRS and ProRS, the anticodon bases of their tRNA substrates are indispensable for aa-tRNA hydrolysis (43, 44). Thus, tRNA selection is achieved *via* the aaRS’s anticodon binding domain, which anchors the tRNA while 3′-end moves from the aminoacylation site to the editing domain (43, 44). In contrast, aa-tRNA deacylation by IleRS and AlaRS relies on D-loop elements on tRNA^Ile^ (45) and a conserved G3:U70 base pair in the acceptor stem of tRNA^Ala^ (46), respectively. The mechanism involving the transfer of tRNA^Ile^ and tRNA^Ala^ to the editing domain after aminoacylation is unknown. Freestanding editing enzymes also exhibit distinct mechanisms of tRNA selection. AlaXp recognizes mischarged tRNA^Ala^ using the same G3:U70 base pair required for editing and aminoacylation by AlaRS (46), although AlaXp-S, an isoform of AlaXp, lacks tRNA specificity (47). Similarly, YbaK, a member of the INS superfamily, lacks inherent tRNA specificity, which results in the deacylation of Cys-tRNA^Pro^ and Cys-tRNA^Cys^ *in vitro* (44, 48). However, *in vivo*, YbaK forms a complex with ProRS that may facilitate the deacylation of Cys-tRNA^Pro^ while preventing Cys-tRNA^Cys^ hydrolysis (49). Broad tRNA specificity is also observed in other freestanding domains from the INS superfamily (38, 39). The broad aa-tRNA specificity of editing enzymes is proposed to protect cells from diverse tRNA aminoacylation errors.

### Transport of aa-tRNAs to the ribosome

After tRNA aminoacylation, EF-Tu carries aa-tRNAs to the A-site of the ribosome (Fig. 2). EF-Tu releases the aa-tRNA cargo once the correct pairing of the tRNA anticodon and the mRNA codon is established, leading to EF-Tu-catalyzed GTP hydrolysis (50, 51, 52, 53, 54). EF-Tu has a uniform binding affinity for all correctly paired aa-tRNA substrates, achieved by binding tRNAs and the amino acid moieties with varying affinity. Thus, the amino acid side chain and the tRNA body contribute thermodynamically to the overall binding affinity to EF-Tu independently (55, 56). In contrast to aaRSs, EF-Tu’s tRNA recognition is mainly determined *via* interactions with the T-stem (57). In the ribosome, the correct pairing of the tRNA anticodon bases with the mRNA is ensured by several features of the anticodon stem-loop, such as the conserved U33, bendability of the anticodon helix, and post-transcriptional modifications (58, 59, 60, 61, 62, 63). The T-arm plays a vital role in the overall tRNA structure and recognition (57, 64, 65). Although EF-Tu has been proposed as a quality control checkpoint during translation since it can reject misacylated tRNAs, little *in vivo* evidence exists. Furthermore, advances in the field of genetic code engineering suggest that EF-Tu’s contribution to the overall translational fidelity is limited or marginal (66).

### mRNA decoding by the ribosome

**Figure 4:**
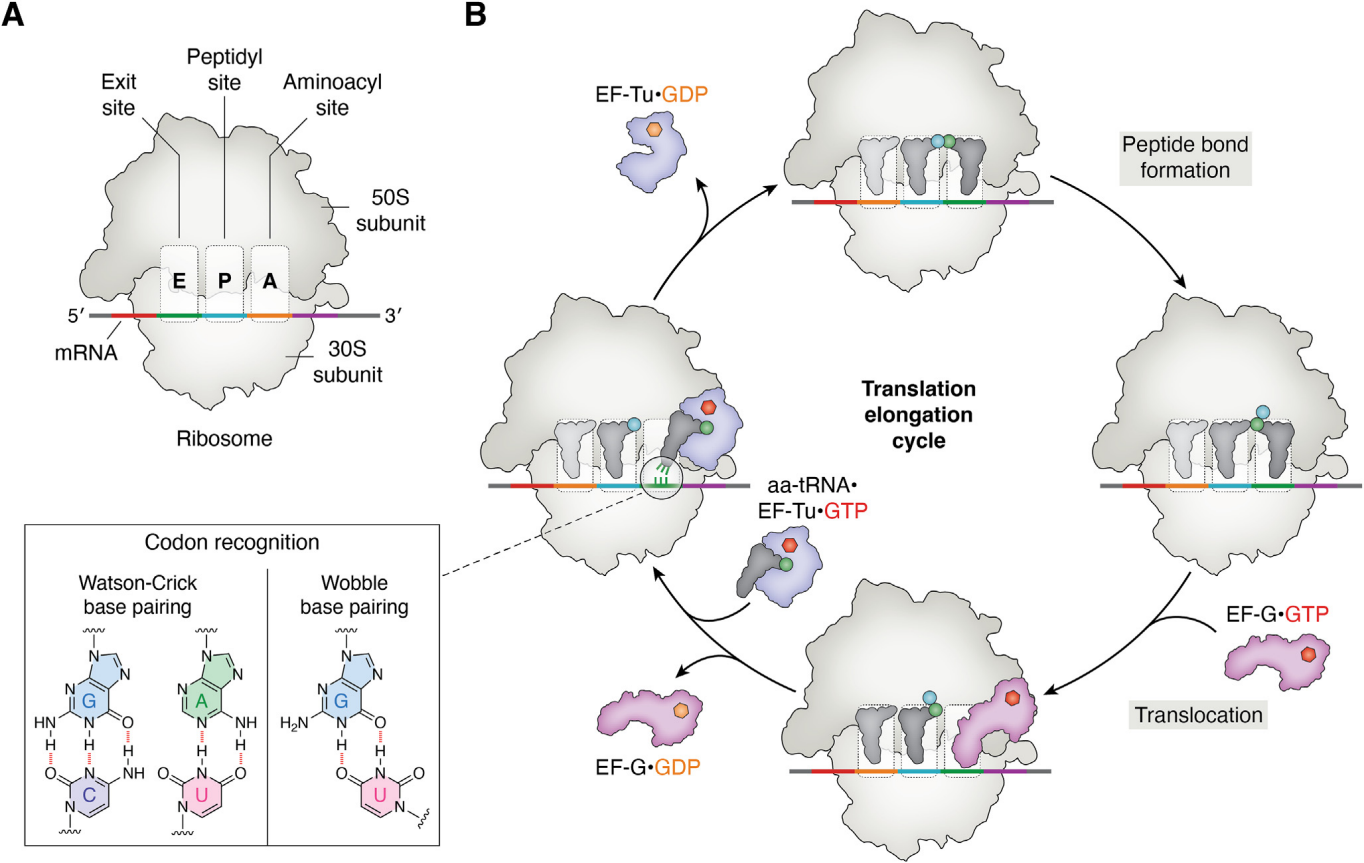
**Translation elongation cycle.***A*, the bacterial ribosome consists of a large (50S) and small (30S) subunit. tRNAs can bind three different cavities called the aminoacyl “A” site, peptidyl “P” site, and the exit “E” site. *B*, during translation elongation, aa-tRNAs are delivered as a complex with EF-Tu•GTP to the ribosome. mRNA codon recognition by the incoming tRNA-anticodon occurs *via* Watson-Crick base pairing and wobble-base pairing. After initial selection, the incoming aa-tRNA accommodates to the A-site, triggering EF-Tu GTP-hydrolysis, releasing it from the ribosome. Subsequently, the tRNA-bound A-site amino acid performs a nucleophilic attack on the tRNA-bound P-site amino acid, resulting in peptide bond formation. EF-G catalyzes the translocation of the mRNA-tRNA complex by one codon, which leaves the A-site idle to bind a new aa-tRNA.

The polymerization of amino acids by the ribosome requires sampling of aa-tRNAs that match the corresponding codons *via* Watson-Crick base pairing as the ribosome advances along the mRNA (Fig. 4). This intricate process requires the ribosome to accurately select the appropriate aa-tRNA from a large pool of different cellular aa-tRNA isoacceptors and isodecoders in cooperation with the EF-Tu (or EF1A) (67). The ribosome actively evaluates the geometry of the codon-anticodon at the first two positions using three universally conserved residues (G530, A1492, and A1493 in the 16S rRNA), allowing wobble base pairing (68, 69, 70, 71, 72). Correct codon-anticodon recognition provokes a coordinated flipping of A1492 and A1493, enabling critical tertiary interactions with G530 (16, 73, 74, 75, 76, 77). This process effectively stabilizes the EF-Tu•GTP•aa-tRNA ternary complex within the A-site. Subsequently, the small ribosomal subunit undergoes a large-scale domain closure (78), promoting the EF-Tu GTPase activation and A-site accommodation of the aa-tRNA (75, 79). In this step, the aa-tRNA is bound in the A/T state, which is transiently distorted and pulls EF-Tu into its GTPase-activated conformation (80, 81, 82, 83, 84, 85). Cognate codon-anticodon interaction induces the efficient conformational change of the decoding center, rapidly triggering the EF-catalyzed GTP hydrolysis (78, 79, 86, 87, 88, 89, 90, 91). While a similar process is possible for near-cognate tRNAs, it is significantly less efficient (78). Most near-cognate tRNAs that induced GTP hydrolysis can still dissociate due to their less stable interaction with the mRNA codon. After EF-Tu•GDP dissociation from the ribosome, the aa-tRNA acceptor stem enters the peptidyl transferase center (PTC) in the large ribosomal subunit, promoting peptide-bond formation between the A-site and P-site tRNAs (92). Precise coordination of the CCA-3′ ends of A- and P-site tRNAs and their rotational movement after peptidyl transfer are crucial to prevent translation errors such as frameshifting or premature termination by ensuring accurate placement of the peptidyl-tRNA in the PTC.

Malfunctioning of any essential component can alter the interaction between the gears of the translational apparatus, impairing translocation mechanics and translational fidelity (93, 94, 95, 96, 97). To prevent mistakes, the ribosome rejects incorrect aa-tRNAs at two stages separated by GTP hydrolysis: initial selection and proofreading. During the initial selection, distinguishing between aa-tRNAs that precisely match the mRNA codon from noncognate aa-tRNAs is challenging, especially for near-cognate aa-tRNAs that differ from the codon at a single position. Translational accuracy at this step is driven by the free energy difference of cognate codon-anticodon recognition and the interaction of A1492, A1493, and G530 (75, 81, 98). Noncognate tRNA results in non-Watson-Crick base pairing, preventing the flipping of A1492 and A1493 and negating the stabilizing role of G530 (16, 75, 99, 100). While energetically unfavorable and kinetically unstable codon-anticodon interactions result in rapid dissociation of noncognate tRNAs, near-cognate tRNAs can form productive Watson-Crick-like conformations and continue to the peptidyl transfer, leading to miscoding (99, 100, 101, 102, 103, 104). The ribosomal error frequency is estimated at 10^−2^ amino acids incorporated (105); however, observed *in vivo* error rates are significantly lower (6, 106, 107, 108, 109, 110) due to the active role of the ribosome during decoding.

### tRNA modifications in aminoacylation and decoding

With an average of 6 to 9 modifications and over 120 known chemically distinct modifications (111, 112, 113), tRNAs are among the most modified cellular RNAs. Anticodon-loop modifications are crucial in mRNA decoding efficiency and accuracy (114). Modifications at position 34 are particularly essential because they enable “wobble” base pairing that facilitates the translation of all 61 codons with fewer tRNAs (63, 114, 115). A prominent example is the critical deamination of A34 to inosine (I34), which expands the base pairing capacity for decoding codons with A, C, or U at the wobble position (115, 116, 117). Additionally, modifications at position 34 are key to ensuring mRNA decoding fidelity. For instance, the lack of 5-methylaminomethyl-2-thiouridine (mnm^5^s^2^) at U34 in bacterial tRNA^Glu^ with UUC anticodon prevents misreading of Gly (GGA and GGG) and Asp (GAU and GAC) codons (110).

Post-transcriptional modifications are also important for ensuring accurate tRNA aminoacylation (16). The lysidine modification at position C34 of *Escherichia coli* tRNA^Ile^ ensures correct recognition by its cognate aaRS while preventing misacylation with Met by MetRS (100). Methylation of *E*. *coli* tRNA^Pro^ G37 contributes to ProRS selection (118). In yeast tRNA^Ile^, the modified wobble base inosine is a positive determinant for aminoacylation by IleRS (119).

## Mechanisms of fortuitous mistranslation

### tRNA mis-aminoacylation

A principal source of mistranslation is the incorrect pairing of amino acids with tRNAs during aminoacylation by aaRSs (2, 120) (Fig. 3). Although aaRSs display high substrate specificity that maintains low error rates during translation, their amino acid selectivity can be compromised by structurally and/or chemically similar amino acids (121). For example, ProRS fails to effectively discriminate against cysteine and alanine (122, 123), PheRS can charge tyrosine onto tRNA^Phe^ (40, 124), ThrRS mistakes serine (125), TyrRS recognizes phenylalanine (126), AlaRS confuses serine and glycine (127), and LeuRS acylates tRNA^Leu^ with isoleucine (128, 129). In addition, amino acids outside the genetic code also challenge aaRSs’ specificity. Non-proteinogenic amino acids are synthesized in dedicated biosynthetic pathways, metabolic byproducts, or by chemical transformation of canonical amino acids due to environmental conditions (130). For instance, Phe and Trp can be transformed into byproducts such as *m*-Tyr and hydroxy-Trp that can be attached to tRNAs by PheRS and TrpRS, respectively (27, 131). Likewise, naturally synthesized amino acids, such as azetidine-2-carboxylic acid (a Pro analog) and β-*N*-methylamino-L-alanine (a Ser analog), are known to infiltrate translation *via* erroneous tRNA aminoacylation (132, 133, 134).

Imbalances in aaRSs and substrate concentration can contribute to aminoacylation errors. For instance, activated human T cells are essential for the adaptive immune system, which helps fight cancer cells by infiltrating their microenvironment and secreting the cytokine interferon-γ (IFNγ). IFNγ secretion triggers the expression of indoleamine 2, 3-dioxygenase 1 (IDO1) in cancer cells, an enzyme that catabolizes Trp to generate metabolites that induce effector T cell dysfunction (135, 136, 137). A recent study demonstrated that IDO1-triggered Trp depletion in cancer cells results in translation of Trp codons with Phe (26). Biochemical and mass spectrometry analyses showed cytosolic TrpRS accepts Phe as a substrate to form mismatched Phe-tRNA^Trp^, which sustains translation of Trp codons and promotes cell viability.

Amino acid substitution in response to specific amino acid deprivation has also been observed in Chinese Hamster Ovary (CHO) cells. In this case, Tyr depletion causes Phe or His incorporation at Tyr codons (138). Misincorporation is the result of tRNA^Tyr^ mischarging with either Phe or His. The biochemical characterization of CHO TyrRS revealed a 25-fold lower specificity for Tyr over Phe than bacterial TyrRS (126, 139). Therefore, CHO appears to have evolved a TyrRS with reduced amino acid specificity relative to *E. coli* TyrRS while developing tolerance to Phe mistranslation. Similarly, low levels of cognate amino acids promote mischarging in many other species (5).

In contrast to amino acids, tRNAs offer a larger surface to interact with aaRSs, increasing their specificity. Although all tRNAs fold into the same L-shape structure with a universally conserved single-stranded CCA-3′ end (Fig. 1*B*), variations in their sequences, especially in the anticodon loop, together with the distinct chemical properties of ribonucleosides generate an RNA code that facilitates tRNA selection by aaRSs. This code involves a set of “identity elements” that are characteristic of each tRNA family and that work as positive (determinants) or negative (anti-determinants) features to establish the productive formation of aaRS-tRNA complexes (16). Most aaRSs gain tRNA specificity using dedicated binding domains that recognize the anticodon sequence. The stringent and complex interactions between tRNAs and aaRSs underscore the importance of ensuring the correct ligation of tRNAs with their cognate amino acids. Despite this, mistakes in tRNA selection still occur due to imbalances in aaRS-tRNA concentrations (140), the absence of tRNA post-transcriptional modifications (100, 141), or modifications in aaRSs that change their tRNA specificity (142, 143).

Mistakes during tRNA aminoacylation are also induced by mutations or chemical modifications of aaRSs or other translation factors. For example, when *E. coli* and *S. cerevisiae* cells experience oxidative stress, a critical Cys residue in ThrRS is oxidized, causing the formation of Ser-tRNA^Thr^ and, ultimately, mistranslation of Thr codons with Ser (22, 144). Also, naturally occurring mutations in clinically isolated variants of *Mycobacterium tuberculosis* GatCAB, the enzyme responsible for the conversion of Asp-tRNA^Asn^ to Asn-tRNA^Asn^ and Glu-tRNA^Gln^ to Gln-tRNA^Gln^, leads to mistranslation of Gln and Asn codons with Glu and Asp, respectively (24, 145).

### Ribosome-promoted mRNA misreading

In contrast to the rare mistranslation by ambiguous codons/tRNAs, mass spectrometry of *E. coli* and yeast proteome revealed frequent specific amino acid substitutions from codon-anticodon mispairing by the ribosome (7). Intriguingly, the errors made by the ribosome are systematic, and certain positions in proteins appear to be more error-prone, while others may even be protected from errors. Notably, these errors occur at sites with higher ribosomal velocity, demonstrating a dilemma between translational speed and fidelity (7). Furthermore, different stress conditions, including amino acid depletion, revealed different amounts, rates, and types of translation error, which suggest that translational errors are central to maintaining cell integrity under different life conditions. Amino acid depletion undermines the quality control during aminoacylation to prevent translational stalling (7).

Mechanistically, ribosomal inaccuracies stem from mRNA and tRNA structures that regulate translational speed (146, 147, 148, 149, 150, 151, 152, 153, 154, 155), ribosome frameshifting, ribosome sliding (97, 153, 154, 156, 157, 158, 159, 160), and noncanonical translation initiation (161, 162). mRNAs that promote high translational speeds lead to an increase in errors due to ineffective rejection of near-cognate tRNAs (163). Similarly, mRNAs with “slippery” sequences wherein tRNAs can base pair with the codon in the −1 or +1-frame can cause ribosomal frameshifting. Frameshifting can occur during translocation when the codon-anticodon interaction is disrupted and the small ribosomal subunit is rearranged. In *E. coli*, EF-G plays a crucial role in translocation and frameshift prevention (164). Spontaneous ribosome frameshifting occurs at a <10^−5^ frequency per codon, resulting in non-functional polypeptides (164). tRNA structural features also induce ribosomal errors. For example, a change in the last base pair of the anticodon stem (32:38) enables the translation of the near-cognate codon GUC by a tRNA^Ala^ (165, 166). A G-to-A mutation at position 24 of tRNA^Trp^’s D-stem allows translation of the UGA stop codon (167, 168, 169). Some naturally occurring mistranslating tRNAs have a rare G33 or A33 (21, 29, 170, 171) that replaces the highly conserved U33 residue within the anticodon loop. U33 plays a critical role in stabilizing a sharp turn to enable the optimal positioning of the anticodon and efficiency decoding (172). The presence of G33 seems to curtail the decoding efficiency of a mistranslating tRNA^Ser^, serving as a mechanism to regulate mistranslation levels (171). The role of A33 in mistranslating tRNAs remains unexplored.

### tRNA modifications in mistranslation

Due to their keystone role in mRNA decoding, tRNA modifications can promote mistranslation. For instance, the mnm^5^s^2^ modification at U34 in tRNA^Lys^ with UUU anticodon results in mistranslation of stop codons and Arg and Asn codons (110). Queuosine is another modification found at position 34 that affects translational fidelity. In bacteria, while conversion of G34 to queuosine in tRNA^Asp^ (GCU anticodon) prevents mistranslation of the Gly codon GGC, the modification in tRNA^Tyr^ (GUA) increases decoding of the Cys codon UGU (110). These antagonistic effects highlight the role of modifications at position N34 during mRNA translation and the complex dynamics of tRNA modifications. In addition to the anticodon bases, modifications of the adjacent base at position 37 are also indispensable for canonical translation. A modified N37, which is almost universally modified with chemically diverse moieties (15, 173), is required for maintaining in-frame decoding and preventing mistranslation (76, 110, 174, 175). Modifications outside of the anticodon branch also impact translational fidelity. For example, the absence of m^5^ at position U54 of human tRNA^Ile^ is associated with the mistranslation of Gln codons with Glu (77).

The specific modification composition of a tRNA depends on environmental conditions and cellular developmental stage due to the dynamic nature of post-transcriptional modifications (160). This dynamism allows the regulation of tRNA activity and provides a facile mechanism to control gene expression at the level of mRNA translation. While tRNA modifications are known to facilitate cellular adaption to different conditions (including infection, immune response, cancer, and stress), less is known about whether mistranslation can be programmed or modulated *via* tRNA modification. Thus, further work examining the biological consequences of the interplay between modification and mistranslation is needed.

## Mechanisms of encoded mistranslation: mistranslating tRNAs

Most known cases of mistranslation are associated with stochastic pairing of amino acids with tRNAs or mismatching of aa-tRNAs with mRNA. However, mistranslation can also be genetically encoded, involving dedicated translation factors or molecular mechanisms (*e.g.*, post-translational modifications) that redefine translation fidelity. Encoded mistranslation contrasts with stochastic mistranslation mainly because the nature of the mistranslation events is genetically programmed. In most cases, the biological role of encoded mistranslation in a particular organism is not well understood. However, encoded mistranslation may offer organisms a molecular tool to overcome physiological and environmental challenges. This section describes examples of genetically encoded tRNAs that enable the natural mistranslating of the genetic code.

### Dual use of tRNAs for Leu mistranslation in yeasts

Many organisms operate noncanonical genetic codes *via* codon reassignment or recoding (176). While codon reassignment leads to the complete change of meaning of a codon, recoding enables codons with dual meanings. Both require the emergence of dedicated translation machinery, particularly of unique tRNAs (176). The first sense-to-sense codon reassignment in a eukaryotic nuclear-encoded genome was identified in the yeast *Candida cylindracea* (103, 177). In *C. cylindracea*, the Leu CUG codon was reassigned to Ser with the emergence of a tRNA^Ser^ with a Leu CAG anticodon and the disappearance of the corresponding tRNA^Leu^ isoacceptor about 272 million years ago (171, 178). This event marked the formation of the 'CUG-Ser1' monophyletic clade of fungi, represented by the *Candida* genus (179), including species of biotechnological and clinical importance (180, 181). Intriguingly, the CUG reassignment to Ser in these organisms also introduced a mechanism of encoded mistranslation caused by the ability of LeuRS to weakly aminoacylate the newly evolved tRNA^Ser^ (CAG) (182). The dual aminoacylation of tRNA^Ser^ (CAG) by SerRS and LeuRS leads to the translation of Ser CUG codons with Ser and Leu. In the opportunistic human pathogen *Candida albicans*, tRNA^Ser^ (CAG)-mediated Leu misincorporation occurs at an estimated rate of 3 to 5% under normal laboratory conditions (182, 183). However, other growth conditions can trigger higher misincorporation rates (up to 28.1%) (183).

Additional reassignments of the CUG Leu codon have also been identified in other yeast species. Species from the genera *Nakazawaea*, *Peterozyma*, and *Pachysolen* are part of the 'CUG-Ala' clade, which encode a tRNA^Ala^ with a CAG anticodon (179, 184, 185). Phylogenetic and bioinformatic analyses also uncovered a novel 'CUG-Ser2' clade consisting of only members of the *Ascoidea* and *Saccharomycopsis* genera. In these species, the tRNA^Ser^ (CAG) contains A37 instead of the m^1^G37 found in the tRNA^Ser^ (CAG) from the 'CUG-Ser1' organisms (179). Consequently, three distinct tRNAs with a CAG anticodon have emerged in each clade (179, 185, 186). The genes for these tRNA mistranslators emerged by duplication of pre-existing tRNA^Ser^ and tRNA^Ala^ genes with the corresponding anticodon mutations. The rise of ambiguous tRNA^Ala^ (CAG) and tRNA^Ser^ (CAG) was likely facilitated by the lack of anticodon recognition by AlaRS and SerRS, respectively, which enabled changes in the anticodon of canonical tRNA^Ala^ and tRNA^Ser^ without preventing aminoacylation by their cognate aaRS (16, 179, 187, 188). Notably, despite the emergence of these novel tRNAs, organisms encoding them did not lose the ability to decode the CUG codon as Leu (179).

Among the three yeast clades with Leu codon reassignment, the CUG-Ser2 clade is unique as the genomes maintained both the tRNA^Ser^ (CAG) and the canonical tRNA^Leu^ (CAG) genes (179, 188). *Ascoidea asiatica* translates CUG codons as Ser and Leu stochastically, while *Saccharomycopsis* species translate CUG only as Ser (179, 189, 190, 191). These results suggest that tRNA^Leu^ (CAG) is not used in translation in *Saccharomycopsis* species. However, recent observations demonstrated that tRNA^Ser^ (CAG) and the canonical tRNA^Leu^ (CAG) are transcribed and aminoacylated in *Saccharomycopsis malanga*, suggesting that these tRNAs are used in translation (191, 192). Based on these opposing observations, the tRNA^Leu^ (CAG) gene is in the process of being eliminated in the CUG-Ser2 clade and will be replaced by the tRNA^Ser^ (CAG) (191). Furthermore, organisms belonging to the CUG-Ser2 clade are in different evolutionary stages, with some species having already lost the purpose for tRNA^Leu^ (CAG) and others not. An alternative possibility is that tRNA^Leu^ (CAG) may be involved in non-translational functions, which would explain why the tRNA is aminoacylated *in vivo* (191, 192) but does not translate CUG codons (179, 189, 190). Additional work is necessary to help understand and clarify the functions and evolution of these tRNAs and the role of mistranslation.

### Bacterial tRNAs with a dual identity for Pro mistranslation

Recent studies uncovered a unique family of bacterial tRNAs with dual identities encoded by soil-borne *Actinomycetes* bacteria, mainly *Streptomyces* and *Kitasatospora* species (21, 29). These tRNAs, collectively known as tRNA^ProX^, combine canonical elements of tRNA^Pro^ with anticodons for Ala (AGC), Asn (AUU), and Thr (AGU). Based on their tRNA^Pro^ aspects, they were named tRNA^ProA^, tRNA^ProN^, and tRNA^ProT^, where the “A”, “N”, and “T” denote their anticodon sequence (Ala, Asn, and Thr, respectively). In addition to their unusual dual identity, tRNA^ProX^ have other rare structural characteristics, including an A33 instead of the almost universally conserved U33, a very unusual A15•A48 pair instead of the common R15•Y48, and G21 instead of the prevalent A21 (21). Moreover, all members of the tRNA^ProX^ family have an A34, which is almost exclusively found in tRNA^Arg^ (ACG) in bacteria (116, 193). A34 of tRNA^Arg^ is post-transcriptionally modified to inosine by the deaminase TadA, expanding the decoding capacity of tRNA^Arg^ to base pair with U, C, or A in the third position of the codon (194). Interestingly, tRNA^ProX^ genes are co-encoded with a unique isoform of bacterial ProRS, known as ProRSx, with an anticodon binding domain that appears to have evolved to interact with the distinct anticodons of tRNA^ProX^. The discovery of ProRSx and the tRNA^ProX^ represents one of the first cases of a devoted aaRS-tRNA pair involved in mistranslation.

Characterization in *E. coli* suggests that ProRSx aminoacylates tRNA^ProX^ with Pro to form Pro-tRNA^ProX^, which is delivered to the ribosome for protein synthesis (21, 29). In *E. coli*, tRNA^ProX^ is also aminoacylated in the absence of ProRSx. *In vitro* experiments showed that *E. coli* ProRS can aminoacylate tRNA^ProX^ despite lacking a Pro anticodon (195), leading to the formation of Pro-tRNA^ProX^. Thus, expression of tRNA^ProA^, tRNA^ProN^, and tRNA^ProT^ in *E. coli* causes mistranslation of Ala, Asn, or Thr codons with Pro, respectively. Interestingly, the physiological impact of tRNA^ProX^ expression drastically varies by the nature of the mistranslated codon. Pro mistranslation of Thr codons is the most detrimental for *E. coli* viability (29). While their initial characterization in *E. coli* has provided key insights, the biological function of ProRSx and tRNA^ProX^ in their host is still unknown. Thus, further studies are needed to understand how and when these genes are expressed and whether mistranslation offers any biological advantage to these bacteria.

### Other bacterial mistranslating tRNAs

Recent large-scale bioinformatics analyses of metagenomic data and bacterial genomes identified several families of rare tRNAs with hybrid identities. For example, strains of the bacterium *Aeromonas salmonicida* encode a selenocysteine (Sec) tRNA (tRNA^Sec^) with Cys GCA anticodon capable of translating targeted Cys UGC codons with Sec (196). In *Desulfotomaculum nigrificans* (and other species), a tRNA^Cys^ (GCA anticodon) with a tRNA^Sec^-like structure decodes Sec UGA codons with Cys (197). This unique tRNA^Cys^ does not interact with canonical EF-Tu. Instead, the Sec-specific elongation factor SelB delivers it to the ribosomes. Additional tRNAs with diverse mismatched identities have also been reported (198). Collectively, the existence of these mistranslating tRNAs highlights the flexibility of the genetic code, contrary to a traditional view of a universal and fixed genetic code.

### Human mistranslating tRNAs

The human genome harbors over 600 predicted tRNA genes, encompassing tRNAs for each proteinogenic amino acid and multiple copies of tRNA isoacceptors and isodecoders (199). The expression and functionality of over 400 of these tRNA genes have been verified (200), displaying differentiated regulation in different human cells and tissues (201, 202, 203). Surprisingly, analyses of the sequenced human genomes have identified several naturally inherited tRNA genes with mismatched or ambiguous identities found in different proportions in the human population (199, 204, 205, 206). Among them are 27 anticodon variants of tRNA^Ala^, tRNA^Ser^, and tRNA^Leu^. 14 tRNAs have unique nonsynonymous anticodon variations (206). These tRNAs pose an increased potential for mistranslation due to the tRNA substrate selection mechanisms of AlaRS, SerRS, and, to a lesser extent, LeuRS, which relies solely on the acceptor stem (16, 187). Thus, nonsynonymous mutations in the anticodon of tRNA^Ala^, tRNA^Ser^, and tRNA^Leu^ potentially cause amino acid mis-incorporation. Notably, one prevalent variant, tRNA^Ala^ with Gly ACC anticodon, is present in over 6% of sequenced individuals (204). AlaRS charges tRNA^Ala^ (ACC), ensuing translation of Gly codons with Ala. Additional in-depth scrutiny of the human genome has unearthed further tRNA genes with anticodon variations potentially inducing mistranslation, such as Thr being mistranslated as Ile, Asp as Val, Val as Ala, Ala as Gly, Cys as Tyr, Arg as Gln, Gly as Stop, Gly as Arg, and, with lesser certainty, Ala as Thr, Cys as Arg, and Ser as Asn (204).

The other 13 tRNAs are synonymous anticodon mutants that, in theory, decode their original “correct” amino acid but at higher or lower efficiencies, potentially altering translation rates (206). The complexity, however, deepens when considering the potential of synonymous anticodon variants becoming mistranslators through nucleotide modification. Deamination of A34 to I34, which allows base pairing with A, C, and U in mRNA (193), could empower tRNAs to mistranslate. For instance, I34 modification of the human tRNA^Asn^ (ATT)-1-1 would lead to Asn incorporation at AAA (Lys) codons (206). A newly discovered tRNA^Ser^ with Phe AAA anticodon, present in 1.8% of the sequenced human population (206), is a substrate for A34-to-I34 editing in eukaryotes (193, 206), resulting in an IAA anticodon that can translate UUU/C (Phe) and UUA (Leu) codons (207, 208).

Besides the ambiguous anticodon variants, cytoplasmic tRNA^Pro^, tRNA^Cys^ (GCA), tRNA^Thr^ (CGT/IGT), tRNA^Gly^ (CCC), and tRNA^Gly^ (GCC) mutants containing a G3:U70 wobble base pair, can lead to misacylation of those tRNAs (24, 205, 209). The G3:U70 wobble base pair is a conserved identity element of tRNA^Ala^, sufficient for AlaRS recognition and aminoacylation (210, 211). Cytosolic AlaRS confuses these non-tRNA^Ala^s with the G:U base pair, ligating them with Ala, eliciting Ala mistranslation.

## Mechanisms of encoded mistranslation: mistranslating aaRSs

Encoded mistranslation involving aaRSs is less intuitive than tRNA-based mistranslation because many aaRSs are inherently error-prone (particularly during amino acid selection). However, in some organisms, aaRSs display naturally increased substrate promiscuity, lack of proofreading activities, or reversibly controlled specificities. The relaxed substrate specificity of these aaRSs can be either constant or temporary, depending on cellular stress conditions. The existence of such aaRSs demonstrates that mistranslation can also be genetically encoded. The examples described below highlight the diverse mechanisms of encoded mistranslation mediated by misacylating aaRSs.

### The malleable tRNA specificity of MetRS

The tRNA specificity of MetRS is determined by a conserved set of elements involving the anticodon bases and the acceptor stem of tRNA^Met^ (16). However, the stringent tRNA specificity can be deliberately relaxed in diverse organisms in response to environmental and stress cues. In mammalian cells exposed to oxidative stress, viruses, or Toll-like receptor ligands, MetRS is post-translationally modified by the extracellular signal-related kinase (ERK1/2) (142, 212). The phosphorylation of MetRS relaxes its tRNA specificity, leading to the acylation of non-tRNA^Met^ with Met, which ultimately causes global mistranslation with Met. MetRS’s tRNA specificity can also be altered by two distinct mechanisms in *E*. *coli* and the archaeon *Aeropyrum pernix*. In *E*. *coli*, succinylation of two Lys residues in the anticodon binding domain endows MetRS with high specificity for tRNA^Met^ (143). However, the modification is removed when *E*. *coli* is grown in anaerobic conditions or in the presence of antibiotics, which triggers global Met acylation of non-Met tRNAs. In hyperthermophilic *A*. *pernix*, the MetRS’s tRNA specificity is affected by changes in environmental temperatures. At the optimal *A*. *pernix* growth temperature of 90 °C, MetRS displays strong selectivity for tRNA^Met^, but it is reduced when cells are grown at 75 °C (213). In contrast to eukaryotic and bacterial MetRS, the relaxed tRNA specificity of the *A*. *pernix* enzyme only leads to acylation of tRNA^Leu^ with Met *via* a yet unknown mechanism (213). This encoded mechanism of Met mistranslation may be a universal feature of MetRS. Nonetheless, additional studies are needed to test this hypothesis and to understand the molecular mechanisms that modulate the changes in tRNA specificity.

### Editing deficient aaRSs

Post-transfer editing aids in preventing the accumulation of mischarged tRNAs and, ultimately, mistranslation. Accordantly, impairment of post-transfer editing can cause physiological defects and can lead to different negative phenotypes. In mice, a mutation in AlaRS that decreases its editing activity by 2-fold provokes the development of ataxia, cardiopathies, and neurological disorders (214, 215). In cultured murine cells, expression of an editing defective ValRS activates caspase-3, prompting apoptosis (216). PheRS with editing deficiency induces apoptosis and reduces cell proliferation, contributing to the development of smaller organ size, mobility impairment, neurodegeneration, and shorter lifespan in *Drosophila melanogaster* (217) and slower growth in yeast (218, 219). In bacteria, defects in the editing domains of PheRS (27, 40), LeuRS (41, 42), IleRS (220), ThrRS (22), and ValRS (221) lead to growth defects caused by cellular dysregulation. Contrary to post-transfer editing, the overall contribution of pre-transfer editing to maintaining translational fidelity in cells is poorly understood. Antagonistic evidence suggests that pre-transfer editing is insufficient to prevent tRNA mischarging in some cases (222) but is required in other cellular environments (223).

Notably, aaRSs with naturally defective post-transfer editing domains exist. Thus, organisms with these aaRSs may be error-prone. Intriguingly, the loss of aaRS post-transfer editing is observed mainly in intracellular or parasitic microorganisms (224). For example, *Microsporidia*, a group of parasitic fungi-like organisms, encode aaRSs with truncated editing domains (25). Consequently, these organisms likely produce diversified proteomes, as observed in the representative microsporidium *Vavraia culicis* (25). Likewise, host-restricted bacteria with small genomes, such as *Mycoplasma*, have high levels of proteomic errors due to their aaRSs with defective editing activity (23, 225). However, the loss of editing domains is not a strict indication that an aaRS becomes a source of translational errors. In some cases, the loss of an editing domain or function is accompanied by increased substrate specificity, as observed for bacterial-type ProRSs lacking the editing domain (226) and mitochondrial PheRS (227).

### Pro mistranslation by ProRSx

The recent discovery of ProRSx is one of the first examples of an aaRS that may have evolved solely for mistranslation of the genetic code. Although ProRSx’s function in its host organisms is unknown, its expression in *E*. *coli* suggests it evolved to exclusively acylate tRNA^ProX^ with Pro (21). Thus, the aminoacylation of canonical tRNA^Pro^ required for protein synthesis in ProRSx-encoding species is expected to be performed by the canonical ProRSs that co-exist with ProRSx. ProRSx is a ProRS isoform found in organisms encoding tRNA^ProX^ genes (discussed in Section 4.2) (21, 29). Based on their phylogenetic relationship and structural homology, ProRSx is predicted to have evolved *via* a duplication of bacterial-type ProRS. One significant difference between the two isoforms is their tRNA anticodon binding domain. ProRSx developed an anticodon binding domain with changes at positions critical for recognizing tRNA anticodon bases 35 and 36. Presumably, these substitutions facilitated the tRNA specificity switch of ProRSx to recognize the tRNA^ProX^ anticodon bases. Furthermore, phylogenetic analyses revealed that members of the ProRSx family also evolved divergently, forming two distinct phylogenetic clades (29). The anticodon identity of the accompanying tRNA^ProX^ gene may have driven the partitioning of the ProRSx family. This suggests that ProRSx encoded with tRNA^ProA^ evolved to interact with the AGC anticodon, whereas ProRSx encoded with tRNA^ProT^ evolved to recognize the AGU anticodon. However, further evidence is needed to support this evolutionary scenario and determine the biological role of ProRSx and mistranslation in host species.

### Bacterial CysRS for Cys mistranslation of Sec codons

An atypical CysRS isoform (named CysRS∗) was found in a subgroup of *Desulfobacterales* bacteria (198). Species encoding CysRS∗ also encode canonical CysRS. In contrast to canonical CysRS, CysRS∗ lacks an anticodon binding domain and has mutations in the region responsible for recognizing the acceptor stem. Although characterization of CysRS∗ is missing, this enzyme is predicted to only ligate Cys to tRNA^Cys^ with UCA anticodon (known as *selC∗* tRNA^Cys^), encoded in the same operon of CysRS∗. The resulting aminoacylation product is known to translate selenocysteine UGA codons with Cys (198).

## The effects of mistranslation

The biological consequences of mistranslation are varied and complex. While mistranslation generally can be harmful to cells, we now know that in several conditions, mistranslation can be employed as a mechanism to overcome unfavorable environmental or physiological changes. An interesting aspect of mistranslation is that the identity of the mistranslated codon(s) and the misincorporated amino acid(s) determine the cellular impact of mistranslation. Thus, each type of mistranslation is different, even unique. For example, Pro-to-Thr mistranslation in *E*. *coli* is substantially more detrimental to cells than Pro-to-Ala or Pro-to-Asn (29). Similarly, in yeast, mistranslation with Ala has varying degrees of toxicity depending on the mistranslated codon (228). The difference in mistranslation tolerance amongst species suggests that the stringency of translational quality control mechanisms has evolved to meet the specific requirements of each species. Different factors may contribute to these differences in cytotoxicity. Presumably, the physicochemical characteristic of the misincorporated amino acid may be a key determinant, while the function of amino acid residues in protein structure and function may be more consequential. For instance, replacing Thr residue with Pro may be more damaging, structurally and catalytically, for an enzyme.

### Negative consenquences

Predictably, mistranslation negatively impacts cell physiology. As discussed in previous Sections of this Review, research in different model organisms have demonstrated that uncontrolled and prolonged mistranslation is toxic, causing diverse phenotypes and death (22, 206, 214, 217, 229, 230, 231, 232, 233). In mice, Ala-to-Ser mistranslation causes severe neurological damage and cardioproteinopathy (214, 215), while Ser-to-Ala and Ser-to-Leu mistranslation increases cell proliferation and tumor growth (233). In human cells, Phe-to-Ser mistranslation by tRNA^Ser^ with AAA anticodon causes increased cytotoxicity and inhibition of protein synthesis, leading to a loss of proteostasis (208). Interestingly, the cytotoxicity is observed in Neuro2a cells but not in HEK 293T cells (205, 206), indicating that mistranslation can have different effects in distinct cell types, which agrees with other studies (214, 215). In contrast, mistranslation of Val codons with the noncanonical amino acid aminobutyric acid causes apoptosis in murine cells (216). In bacteria, mistranslation can cause significant growth defects. Mistranslation of Ile codons with the noncanonical amino acid norvaline leads to severe growth arrest in *E*. *coli* (42, 231). Similarly, mistranslation of Phe codons with *m*-Tyr or Ala codons with Ser inhibits *E*. *coli* growth (27, 234). The cases described above only represent a fraction of all known cases involving mistranslation, but they exemplify the overall negative consequences of mistranslation.

### Adaptive advantage

Given the deleterious effects of mistranslation and the multiple translation quality control checkpoints in place, the notion of mistranslation as a practical mechanism for survival is paradoxical. However, accumulating evidence shows that organisms cannot only tolerate high degrees of mistranslation but can also be used to counter and resist cellular stresses such as nutritional scarcity, antibiotic exposure, or chemical and immunological challenges (5, 8, 26, 30, 142, 182, 235, 236, 237, 238, 239, 240, 241, 242, 243, 244, 245, 246, 247, 248). Thus, negative regulation of translation fidelity to increase translational errors may serve as a bet-hedging strategy in which cells trade off the fitness cost of mistranslation to respond to a harmful environmental change (249). In these cases, mistranslation is conditional and transient, triggered by a particular environmental cue. For example, exposure to oxidative stress prompts global mistranslation of different codons with Met in mammalian cells (142, 212). Given the antioxidant properties of Met, mistranslation may protect proteins from oxidative damage by scavenging free reactive oxygen species and forming reversible Met sulfoxide on the surface or near active sites of proteins (250, 251). Met mistranslation in response to oxidative stress also occurs in yeast (252) and *E. coli* (143, 239). Other conditions, such as viral infections, temperature changes, antibiotic exposure, and anaerobiosis, also promote Met mistranslation (142, 143, 213), although it is unclear how Met can shield cells in these conditions.

Ribosome-promoted mistranslation can also protect bacteria against oxidative (236) and heat stress (253) by activating stress response pathways that help maintain cellular proteostasis. Increased ribosomal errors caused by an I119N mutation in the ribosomal protein RpsD (254) upregulates the expression of RpoS, the general stress response sigma factor, which activates the sigma factor 32 (RpoH) *via* an unknown mechanism. RpoH promotes the synthesis of proteases and chaperones (*e.g.*, DnaK and GroEL) to restore and preserve proteostasis in *E*. *coli* cells expressing RpsD I119N (253). Thus, the activation of the general stress response due to moderate levels of mistranslation can predispose cells to resist heat (253) and oxidative stress (236). In *S*. *cerevisiae,* increased mistranslation contributes to cadmium resistance (255).

Other types of mistranslation have also been shown to increase antibiotic tolerance. Asp-to-Asn and Glu-to-Gln mistranslation endows *Mycobacteria* with increased resistance to rifampicin (24, 256), and Pro mistranslation of Ala and Asn codons protects *E*. *coli* from ampicillin or carbenicillin (29). Similarly, mistranslation due to promiscuous initiation at non-AUG codons or by the presence of non-proteinogenic amino acids increases resistance against ciprofloxacin (240), while an error-prone ribosome mutant helps cells tolerate low concentrations of cefotaxime (257). Increased antibiotic resistance is also observed in the fungal human pathogen *C*. *albicans* against fluconazole (258). Together, these studies suggest that mistranslation may be a *bona fide* mechanism of antibiotic tolerance (259).

Imbalances in amino acid metabolism can stall protein synthesis, which challenges cell viability. When the concentration of a particular amino acid is limiting, the corresponding aaRS can accept a near-cognate amino acid and attach it to cognate tRNA. While the resulting mischarged tRNA causes mistranslation, it affords protein synthesis and survival. A prominent demonstration was the total replacement of Trp with the synthetic amino acid analog L-β-(thieno[3,2-b]pyrrolyl)alanine ([3,2]Tpa) in *E*. *coli* (260). This synthetic amino acid is aminoacylated by TrpRS in the absence of Trp, enabling translation of Trp codons during sustaining protein synthesis. A similar but natural phenomenon occurs in human cancer cells, in which proteome-wide Trp-to-Phe substitutions arise from Trp depletion by IFNγ-mediated IDO1 induction (26). In this low Trp condition, cells can prolong their survival using Phe. Moreover, Trp-to-Phe containing peptides produced by the cancer cells are presented by Human Leucocyte Antigen (HLA) class 1 on the cell surface. Enriching Trp-to-Phe substituents by cancer cells is associated with increased immune reactivity and oncogenic signaling (26).

Recent studies have begun to provide insights that help explain how mistranslation can be advantageous in a particular condition. For example, activation of stress response pathways induced by mistranslation can aid cells in countering conditions in which these pathways play a critical role in survival (236, 253). Despite this progress, our overall knowledge of the mechanisms by which mistranslation provides an advantage to an organism is significantly limited.

## Outlook

The recent discoveries of noncanonical interpretations of the genetic code underscore the dynamic nature of the code and how organisms have altered the rules to adapt to diverse lifestyles (192, 261, 262). At the center of these exciting discoveries are tRNAs, which, after more than 60 years of their discovery (263), remain at the forefront of molecular biology due to their complex biology that extends beyond translation (1). With the rapidly increasing number of sequenced genomes and the vast number already available, more organisms with novel genetic code rules are likely to be discovered, which can be facilitated by identifying novel tRNAs with unique features. Identifying new tRNAs can be enabled by developing and implementing enhanced algorithms and trained artificial intelligence (AI) systems. AI may also be well-suited for distinguishing misannotated noncanonical tRNAs from the canonical ones in various databases (19, 264, 265, 266, 267, 268), paving the way for further in-depth exploration. The AI-assisted tools can support the advances in high-throughput sequencing methods, accelerating the discovery of novel noncanonical components of the translational machinery, as 90% of cellular life on earth is unknown (269). The computational identification and annotation of such tRNA genes will accelerate their functional characterization, which may open the door to discovering exciting new functions and biological processes, such as tRNA modifications, modes of mRNA decoding, new aaRSs, and non-translational roles.

In the context of mistranslation, establishing how tRNAs regulate translational fidelity can shed light on how organisms can leverage translational errors as an adaptive mechanism to cope with adverse or suboptimal growth conditions. While we know mistranslating tRNAs exist, we know little about how their host organisms use them. Thus, defining their expression profile and regulation may constitute an avenue to better understand the biology of these tRNAs. Moreover, determining the role of post-transcriptional modifications in translational fidelity could also provide insights into mistranslation. Particularly, studies focusing on how the expression of tRNA-modifying enzymes is regulated in different conditions and how it can impact tRNA decoding capacity and fidelity can help fill existing knowledge gaps. Achieving these goals will require developing and implementing sensitive, reproducible, and inexpensive reporter-based and mass spectrometry methods to identify subtle and highly specific mistranslation events. Furthermore, the natural scenarios in which mistranslation is biologically consequential (negatively or positively) need to be investigated, as most of our current understanding of mistranslation is based on synthetic mistranslation systems responding to artificial conditions.

## Conflicts of interest

The authors declare no conflict of interest.
